# Aqua­(3-formyl-2-oxidobenzoato-κ^2^ *O* ^1^,*O* ^2^)(1,10-phenanthroline-κ^2^ *N*,*N*′)copper(II) dimethyl­formamide solvate

**DOI:** 10.1107/S1600536809011659

**Published:** 2009-04-08

**Authors:** Zhao-Wen Yu, Ling Chang, Peng Song, Min-Hui He

**Affiliations:** a Institute of Molecular and Crystal Engineering, College of Chemistry and Chemical Engineering, Henan University, Kaifeng 475001, Henan, People’s Republic of China

## Abstract

In the structure of the title complex, [Cu(C_8_H_4_O_4_)(C_12_H_8_N_2_)(H_2_O)]·C_3_H_7_NO, the Cu^II^ ion is penta­coordinated in a distorted square-pyramidal geometry by two O atoms of a 3-formyl-2-oxidobenzoate dianion and two N atoms of a 1,10-phenanthroline ligand occupying the basal plane and a water O atom located at the apical site. The structure displays O—H⋯O hydrogen bonding and inter­molecular π–π stacking inter­actions between 1,10-phenantroline ligands [inter­planar distance of 3.448 (5) Å].

## Related literature

For the structure of the methanol solvate of aqua­(3-formyl-2-oxidobenzoato-κ^2^ *O* ^1^,*O* ^2^)(1,10-phenanthroline-κ^2^ *N*,*N*′)copper(II), see: Zhang *et al.* (2008 ▶).
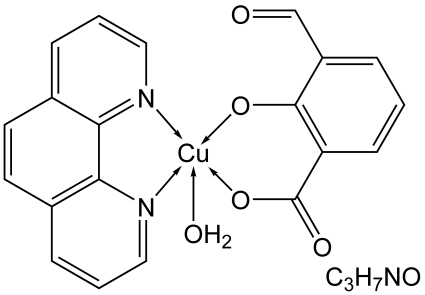


## Experimental

### 

#### Crystal data

[Cu(C_8_H_4_O_4_)(C_12_H_8_N_2_)(H_2_O)]·C_3_H_7_NO
*M* *_r_* = 498.97
Triclinic, 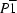

*a* = 9.6936 (6) Å
*b* = 10.9020 (12) Å
*c* = 11.2800 (7) Å
α = 103.834 (1)°
β = 109.764 (1)°
γ = 98.604 (1)°
*V* = 1054.09 (15) Å^3^
*Z* = 2
Mo *K*α radiation
μ = 1.08 mm^−1^
*T* = 296 K
0.39 × 0.35 × 0.28 mm

#### Data collection

Bruker SMART APEXII CCD area-detector diffractometer
Absorption correction: multi-scan (*SADABS*; Bruker, 2005 ▶) *T*_min_ = 0.677, *T*_max_ = 0.751
5440 measured reflections
3687 independent reflections
3452 reflections with *I* > 2σ(*I*)
*R*_int_ = 0.011

#### Refinement

*R*[*F* ^2^ > 2σ(*F* ^2^)] = 0.036
*wR*(*F* ^2^) = 0.112
*S* = 1.08
3687 reflections
301 parameters
H-atom parameters constrained
Δρ_max_ = 0.81 e Å^−3^
Δρ_min_ = −0.48 e Å^−3^

### 

Data collection: *APEX2* (Bruker, 2005 ▶); cell refinement: *SAINT* (Bruker, 2005 ▶); data reduction: *SAINT*; program(s) used to solve structure: *SHELXS97* (Sheldrick, 2008 ▶); program(s) used to refine structure: *SHELXL97* (Sheldrick, 2008 ▶); molecular graphics: *SHELXTL* (Sheldrick, 2008 ▶); software used to prepare material for publication: *SHELXTL*.

## Supplementary Material

Crystal structure: contains datablocks I, global. DOI: 10.1107/S1600536809011659/gk2199sup1.cif

Structure factors: contains datablocks I. DOI: 10.1107/S1600536809011659/gk2199Isup2.hkl

Additional supplementary materials:  crystallographic information; 3D view; checkCIF report

## Figures and Tables

**Table 1:** Hydrogen-bond geometry (Å, °)

*D*—H⋯*A*	*D*—H	H⋯*A*	*D*⋯*A*	*D*—H⋯*A*
O1*W*—H1*WB*⋯O4^i^	0.85	1.91	2.741 (3)	167
O1*W*—H1*WA*⋯O5	0.85	1.96	2.794 (3)	167

Symmetry code: (i) 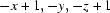.

**Fig. 1.**
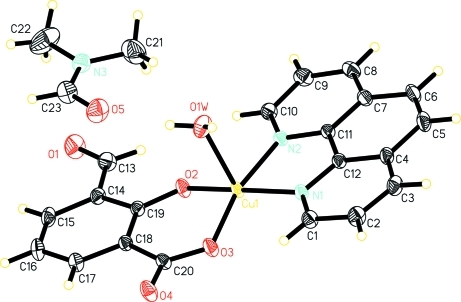
The molecular structure of the title compound, showing the atom-labelling scheme. Displacement ellipsoides are shown at the 25% probability level.

**Fig. 2.**
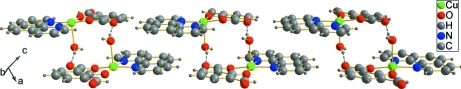
The molecular packing of the title compound. Hydrogen bonds are indicated by dashed lines.

## supplementary crystallographic information

### Comment

Recently we have reported the crystal structure of the methanol solvate of the title coordination compound. Here we report the crystal structure of its dimethylformamide solvate.

In the complex, the Cu^2+^ ion is pentacoordinated, with two O atoms of 3-carboxylsalicylaldehyde anion and two N atoms  from 1,10-phenanthroline ligand in the basal plane and the O atom of water molecule completing the square-pyramidal geometry from the apical site (Fig. 1). The atoms N1, N2, O3 and O2 are nearly coplanar, and the Cu atom is displaced by 0.137 Å from this plane towards the apical O atom, giving the N1–Cu1–O2 angle of 172.36 (8)° and N2–Cu1–O3 angle of 166.78 (9) °. The structure of the complex molecule is very similar to that observed in the methanol solvate (Zhang *et al.*, 2008).

There are two kinds of intermolecular hydrogen bonds in the crystal. One is between the H1WA atom of the water molecule and the O5 atom of  the DMF molecule and the other is between the H1WB atom of the water molecule and the uncoordinated O4 atom (O4^i^: (i) = -*x* + 1, -*y*, -*z* + 1) of the carboxylate group. Intermolecular hydrogen bonds and  π–π stacking interactions phenanthroline ligands (the interplanar distance of 3.448 Å) generate one-dimensional structure shown in Fig. 2.

### Experimental

3-Carboxylsalicylaldehyde (0.166 g, 1.0 mmol) was dissolved in 10 ml of aqueous solution containing 0.080 g  (2.0 mmol) NaOH. To this solution, 15 ml of DMF solution containing 1,10-phenanthroline (0.1982 g, 1 mmol) and CuCl_2_.2H_2_O (0.1705 g, 1 mmol) was added. The mixture was stirred at room temperature for 2 h, then filtered to give a green solution. The filtrate was airproofed and kept at room temperature. Two weeks later, green block-shaped crystal of X-ray quality were obtained.

### Refinement

The positions of the water H atoms obtained from a difference Fourier map were constraestained to ideal water geometry and fixed in the final stages of refinement (O-H 0.85 Å). All other H atoms were included in calculated positions, with C—H distances ranging from 0.93 to 0.96 Å. They were refined in the riding-model approximation, with *U*_iso_(H) = 1.2 *U*_eq_ (C) or 1.5 *U*_eq_(C, O).

### Crystal data

**Table:**

[Cu(C_8_H_4_O_4_)(C_12_H_8_N_2_)(H_2_O)]·C_3_H_7_NO	*Z* = 2
*M**_r_* = 498.97	*F*(000) = 514
Triclinic, *P*1	*D*_x_ = 1.572 Mg m^−^^3^
Hall symbol: -P 1	Mo *K*α radiation, λ = 0.71073 Å
*a* = 9.6936 (6) Å	Cell parameters from 4490 reflections
*b* = 10.9020 (12) Å	θ = 2.3–28.3°
*c* = 11.2800 (7) Å	µ = 1.08 mm^−^^1^
α = 103.834 (1)°	*T* = 296 K
β = 109.764 (1)°	Block, green
γ = 98.604 (1)°	0.39 × 0.35 × 0.28 mm
*V* = 1054.09 (15) Å^3^	

### Data collection

**Table:**

Bruker SMART APEXII CCD area-detector diffractometer	3687 independent reflections
Radiation source: fine-focus sealed tube	3452 reflections with *I* > 2σ(*I*)
graphite	*R*_int_ = 0.011
φ and ω scans	θ_max_ = 25.0°, θ_min_ = 2.0°
Absorption correction: multi-scan (*SADABS*; Bruker, 2005)	*h* = −11→11
*T*_min_ = 0.677, *T*_max_ = 0.751	*k* = −12→12
5440 measured reflections	*l* = −13→5

### Refinement

**Table:**

Refinement on *F*^2^	Primary atom site location: structure-invariant direct methods
Least-squares matrix: full	Secondary atom site location: difference Fourier map
*R*[*F*^2^ > 2σ(*F*^2^)] = 0.036	Hydrogen site location: inferred from neighbouring sites
*wR*(*F*^2^) = 0.112	H-atom parameters constrained
*S* = 1.08	*w* = 1/[σ^2^(*F*_o_^2^) + (0.0719*P*)^2^ + 0.6371*P*] where *P* = (*F*_o_^2^ + 2*F*_c_^2^)/3
3687 reflections	(Δ/σ)_max_ < 0.001
301 parameters	Δρ_max_ = 0.81 e Å^−^^3^
0 restraints	Δρ_min_ = −0.47 e Å^−^^3^
0 constraints	

### Special details

**Table:**

Geometry. All e.s.d.'s (except the e.s.d. in the dihedral angle between two l.s. planes) are estimated using the full covariance matrix. The cell e.s.d.'s are taken into account individually in the estimation of e.s.d.'s in distances, angles and torsion angles; correlations between e.s.d.'s in cell parameters are only used when they are defined by crystal symmetry. An approximate (isotropic) treatment of cell e.s.d.'s is used for estimating e.s.d.'s involving l.s. planes.
Refinement. Refinement of *F*^2^ against ALL reflections. The weighted *R*-factor *wR* and goodness of fit *S* are based on *F*^2^, conventional *R*-factors *R* are based on *F*, with *F* set to zero for negative *F*^2^. The threshold expression of *F*^2^ > σ(*F*^2^) is used only for calculating *R*-factors(gt) *etc*. and is not relevant to the choice of reflections for refinement. *R*-factors based on *F*^2^ are statistically about twice as large as those based on *F*, and *R*- factors based on ALL data will be even larger.

### Fractional atomic coordinates and isotropic or equivalent isotropic displacement parameters (Å^2^)

**Table:**

	*x*	*y*	*z*	*U*_iso_*/*U*_eq_	
Cu1	0.16328 (3)	0.00556 (3)	0.42283 (3)	0.03264 (14)	
O1W	0.3462 (2)	0.1436 (2)	0.3911 (2)	0.0530 (5)	
H1WA	0.3993	0.2098	0.4581	0.064*	
H1WB	0.4065	0.1074	0.3640	0.064*	
N1	0.1623 (2)	−0.1462 (2)	0.2786 (2)	0.0327 (4)	
N2	−0.0089 (2)	0.0216 (2)	0.2678 (2)	0.0308 (4)	
O1	0.0712 (3)	0.4672 (2)	0.7470 (2)	0.0664 (7)	
O2	0.1357 (2)	0.13957 (18)	0.54916 (17)	0.0398 (4)	
O3	0.3000 (2)	−0.0508 (2)	0.55253 (18)	0.0448 (5)	
O4	0.4950 (2)	−0.0174 (2)	0.73640 (19)	0.0488 (5)	
C1	0.2529 (3)	−0.2267 (3)	0.2873 (3)	0.0398 (6)	
H1	0.3279	−0.2160	0.3693	0.048*	
C2	0.2386 (4)	−0.3274 (3)	0.1765 (3)	0.0464 (7)	
H2	0.3035	−0.3824	0.1859	0.056*	
C3	0.1309 (3)	−0.3448 (3)	0.0559 (3)	0.0443 (6)	
H3	0.1210	−0.4118	−0.0176	0.053*	
C4	0.0332 (3)	−0.2596 (2)	0.0429 (2)	0.0359 (6)	
C5	−0.0824 (3)	−0.2675 (3)	−0.0794 (3)	0.0459 (7)	
H5	−0.0983	−0.3331	−0.1561	0.055*	
C6	−0.1692 (3)	−0.1811 (3)	−0.0857 (3)	0.0449 (7)	
H6	−0.2427	−0.1875	−0.1669	0.054*	
C7	−0.1500 (3)	−0.0799 (3)	0.0307 (2)	0.0364 (6)	
C8	−0.2360 (3)	0.0125 (3)	0.0314 (3)	0.0445 (6)	
H8	−0.3119	0.0107	−0.0465	0.053*	
C9	−0.2064 (3)	0.1058 (3)	0.1490 (3)	0.0460 (7)	
H9	−0.2626	0.1677	0.1510	0.055*	
C10	−0.0924 (3)	0.1080 (3)	0.2656 (3)	0.0376 (6)	
H10	−0.0742	0.1719	0.3442	0.045*	
C11	−0.0379 (3)	−0.0707 (2)	0.1520 (2)	0.0302 (5)	
C12	0.0550 (3)	−0.1618 (2)	0.1578 (2)	0.0307 (5)	
C13	0.0912 (4)	0.3653 (3)	0.6920 (3)	0.0460 (7)	
H13	0.0230	0.3205	0.6056	0.055*	
C14	0.2124 (3)	0.3076 (2)	0.7494 (3)	0.0353 (6)	
C15	0.3105 (3)	0.3681 (3)	0.8826 (3)	0.0432 (6)	
H15	0.2961	0.4433	0.9314	0.052*	
C16	0.4270 (4)	0.3178 (3)	0.9413 (3)	0.0503 (7)	
H16	0.4925	0.3591	1.0292	0.060*	
C17	0.4463 (3)	0.2053 (3)	0.8687 (3)	0.0413 (6)	
H17	0.5252	0.1713	0.9100	0.050*	
C18	0.3532 (3)	0.1408 (2)	0.7370 (2)	0.0314 (5)	
C19	0.2317 (3)	0.1921 (2)	0.6732 (2)	0.0308 (5)	
C20	0.3865 (3)	0.0178 (3)	0.6722 (2)	0.0340 (5)	
C21	0.2290 (5)	0.4426 (5)	0.4552 (4)	0.0845 (12)	
H21A	0.2696	0.3733	0.4216	0.127*	
H21B	0.2083	0.4936	0.3956	0.127*	
H21C	0.1370	0.4063	0.4626	0.127*	
C22	0.3128 (7)	0.6469 (4)	0.6359 (6)	0.1041 (18)	
H22A	0.4044	0.6995	0.7088	0.156*	
H22B	0.2323	0.6350	0.6666	0.156*	
H22C	0.2869	0.6897	0.5692	0.156*	
N3	0.3350 (3)	0.5228 (3)	0.5809 (2)	0.0490 (6)	
C23	0.4569 (4)	0.4849 (4)	0.6420 (4)	0.0633 (9)	
H23	0.5258	0.5400	0.7245	0.076*	
O5	0.4840 (3)	0.3805 (2)	0.5953 (3)	0.0700 (7)	

### Atomic displacement parameters (Å^2^)

**Table:**

	*U*^11^	*U*^22^	*U*^33^	*U*^12^	*U*^13^	*U*^23^
Cu1	0.0417 (2)	0.0314 (2)	0.02085 (19)	0.01603 (14)	0.00811 (14)	0.00282 (13)
O1W	0.0483 (11)	0.0443 (11)	0.0639 (14)	0.0135 (9)	0.0265 (10)	0.0044 (10)
N1	0.0378 (11)	0.0313 (11)	0.0256 (10)	0.0106 (9)	0.0101 (9)	0.0051 (8)
N2	0.0345 (10)	0.0324 (10)	0.0259 (10)	0.0096 (8)	0.0121 (8)	0.0084 (8)
O1	0.0850 (17)	0.0556 (14)	0.0610 (14)	0.0422 (13)	0.0300 (13)	0.0051 (11)
O2	0.0464 (10)	0.0414 (10)	0.0252 (9)	0.0210 (8)	0.0080 (8)	0.0017 (7)
O3	0.0598 (12)	0.0410 (10)	0.0261 (9)	0.0268 (9)	0.0061 (8)	0.0038 (8)
O4	0.0499 (11)	0.0577 (12)	0.0329 (10)	0.0296 (10)	0.0074 (9)	0.0066 (9)
C1	0.0441 (14)	0.0377 (14)	0.0369 (14)	0.0175 (12)	0.0148 (12)	0.0074 (11)
C2	0.0518 (17)	0.0425 (15)	0.0467 (16)	0.0213 (13)	0.0221 (14)	0.0070 (13)
C3	0.0533 (16)	0.0370 (14)	0.0407 (15)	0.0119 (12)	0.0244 (13)	−0.0006 (12)
C4	0.0425 (14)	0.0337 (13)	0.0271 (12)	0.0047 (11)	0.0156 (11)	0.0017 (10)
C5	0.0525 (16)	0.0460 (16)	0.0281 (13)	0.0048 (13)	0.0151 (12)	−0.0024 (12)
C6	0.0450 (15)	0.0537 (17)	0.0234 (12)	0.0048 (13)	0.0049 (11)	0.0067 (12)
C7	0.0355 (13)	0.0408 (14)	0.0286 (12)	0.0043 (11)	0.0102 (10)	0.0100 (11)
C8	0.0400 (14)	0.0555 (17)	0.0362 (14)	0.0144 (13)	0.0077 (11)	0.0200 (13)
C9	0.0469 (16)	0.0496 (17)	0.0485 (17)	0.0248 (13)	0.0182 (13)	0.0203 (14)
C10	0.0422 (14)	0.0361 (13)	0.0363 (14)	0.0142 (11)	0.0171 (11)	0.0092 (11)
C11	0.0326 (12)	0.0316 (12)	0.0255 (11)	0.0061 (10)	0.0121 (10)	0.0077 (10)
C12	0.0333 (12)	0.0320 (12)	0.0262 (12)	0.0062 (10)	0.0133 (10)	0.0069 (10)
C13	0.0571 (17)	0.0447 (16)	0.0430 (16)	0.0221 (13)	0.0259 (14)	0.0105 (13)
C14	0.0421 (14)	0.0314 (13)	0.0337 (13)	0.0072 (11)	0.0204 (11)	0.0053 (10)
C15	0.0521 (16)	0.0328 (13)	0.0371 (14)	0.0061 (12)	0.0198 (12)	−0.0034 (11)
C16	0.0530 (17)	0.0463 (16)	0.0326 (14)	0.0055 (13)	0.0094 (13)	−0.0063 (12)
C17	0.0402 (14)	0.0431 (15)	0.0329 (14)	0.0097 (12)	0.0101 (11)	0.0047 (12)
C18	0.0348 (12)	0.0314 (12)	0.0265 (12)	0.0057 (10)	0.0131 (10)	0.0057 (10)
C19	0.0353 (12)	0.0303 (12)	0.0271 (12)	0.0061 (10)	0.0156 (10)	0.0055 (10)
C20	0.0388 (13)	0.0391 (14)	0.0254 (12)	0.0138 (11)	0.0129 (11)	0.0092 (11)
C21	0.072 (3)	0.098 (3)	0.073 (3)	0.031 (2)	0.010 (2)	0.031 (2)
C22	0.140 (5)	0.056 (2)	0.152 (5)	0.046 (3)	0.091 (4)	0.033 (3)
N3	0.0601 (16)	0.0423 (13)	0.0492 (15)	0.0193 (11)	0.0253 (13)	0.0121 (11)
C23	0.062 (2)	0.065 (2)	0.056 (2)	0.0156 (18)	0.0195 (17)	0.0136 (17)
O5	0.0769 (17)	0.0578 (15)	0.0774 (17)	0.0293 (13)	0.0319 (14)	0.0145 (13)

### Geometric parameters (Å, °)

**Table:**

Cu1—O2	1.9012 (18)	C8—C9	1.374 (4)
Cu1—O3	1.9071 (18)	C8—H8	0.9300
Cu1—N1	2.020 (2)	C9—C10	1.394 (4)
Cu1—N2	2.033 (2)	C9—H9	0.9300
Cu1—O1W	2.329 (2)	C10—H10	0.9300
O1W—H1WA	0.8500	C11—C12	1.435 (4)
O1W—H1WB	0.8500	C13—C14	1.448 (4)
N1—C1	1.328 (3)	C13—H13	0.9300
N1—C12	1.359 (3)	C14—C15	1.403 (4)
N2—C10	1.330 (3)	C14—C19	1.421 (3)
N2—C11	1.356 (3)	C15—C16	1.366 (5)
O1—C13	1.215 (4)	C15—H15	0.9300
O2—C19	1.315 (3)	C16—C17	1.379 (4)
O3—C20	1.284 (3)	C16—H16	0.9300
O4—C20	1.231 (3)	C17—C18	1.386 (4)
C1—C2	1.403 (4)	C17—H17	0.9300
C1—H1	0.9300	C18—C19	1.426 (4)
C2—C3	1.354 (4)	C18—C20	1.502 (3)
C2—H2	0.9300	C21—N3	1.408 (5)
C3—C4	1.420 (4)	C21—H21A	0.9600
C3—H3	0.9300	C21—H21B	0.9600
C4—C12	1.395 (3)	C21—H21C	0.9600
C4—C5	1.427 (4)	C22—N3	1.428 (5)
C5—C6	1.352 (4)	C22—H22A	0.9600
C5—H5	0.9300	C22—H22B	0.9600
C6—C7	1.434 (4)	C22—H22C	0.9600
C6—H6	0.9300	N3—C23	1.332 (5)
C7—C11	1.400 (3)	C23—O5	1.240 (4)
C7—C8	1.401 (4)	C23—H23	0.9300
			
O2—Cu1—O3	94.58 (8)	C9—C10—H10	119.0
O2—Cu1—N1	172.36 (8)	N2—C11—C7	123.8 (2)
O3—Cu1—N1	89.63 (8)	N2—C11—C12	116.4 (2)
O2—Cu1—N2	93.28 (8)	C7—C11—C12	119.8 (2)
O3—Cu1—N2	166.80 (9)	N1—C12—C4	123.4 (2)
N1—Cu1—N2	81.45 (8)	N1—C12—C11	116.5 (2)
O2—Cu1—O1W	95.02 (8)	C4—C12—C11	120.1 (2)
O3—Cu1—O1W	96.84 (9)	O1—C13—C14	125.5 (3)
N1—Cu1—O1W	90.80 (8)	O1—C13—H13	117.2
N2—Cu1—O1W	93.03 (8)	C14—C13—H13	117.2
Cu1—O1W—H1WA	114.5	C15—C14—C19	120.6 (3)
Cu1—O1W—H1WB	115.6	C15—C14—C13	118.5 (2)
H1WA—O1W—H1WB	107.7	C19—C14—C13	121.0 (2)
C1—N1—C12	118.3 (2)	C16—C15—C14	120.7 (3)
C1—N1—Cu1	128.79 (18)	C16—C15—H15	119.6
C12—N1—Cu1	112.92 (16)	C14—C15—H15	119.6
C10—N2—C11	117.8 (2)	C15—C16—C17	119.3 (3)
C10—N2—Cu1	129.47 (18)	C15—C16—H16	120.3
C11—N2—Cu1	112.70 (16)	C17—C16—H16	120.3
C19—O2—Cu1	123.98 (16)	C16—C17—C18	122.8 (3)
C20—O3—Cu1	126.98 (17)	C16—C17—H17	118.6
N1—C1—C2	121.9 (3)	C18—C17—H17	118.6
N1—C1—H1	119.1	C17—C18—C19	118.9 (2)
C2—C1—H1	119.1	C17—C18—C20	116.5 (2)
C3—C2—C1	120.3 (3)	C19—C18—C20	124.5 (2)
C3—C2—H2	119.8	O2—C19—C14	117.8 (2)
C1—C2—H2	119.8	O2—C19—C18	124.5 (2)
C2—C3—C4	119.2 (2)	C14—C19—C18	117.7 (2)
C2—C3—H3	120.4	O4—C20—O3	120.9 (2)
C4—C3—H3	120.4	O4—C20—C18	119.2 (2)
C12—C4—C3	116.9 (2)	O3—C20—C18	119.9 (2)
C12—C4—C5	119.1 (2)	N3—C21—H21A	109.5
C3—C4—C5	124.0 (2)	N3—C21—H21B	109.5
C6—C5—C4	121.1 (2)	H21A—C21—H21B	109.5
C6—C5—H5	119.5	N3—C21—H21C	109.5
C4—C5—H5	119.5	H21A—C21—H21C	109.5
C5—C6—C7	121.1 (2)	H21B—C21—H21C	109.5
C5—C6—H6	119.4	N3—C22—H22A	109.5
C7—C6—H6	119.4	N3—C22—H22B	109.5
C11—C7—C8	117.1 (2)	H22A—C22—H22B	109.5
C11—C7—C6	118.8 (2)	N3—C22—H22C	109.5
C8—C7—C6	124.1 (2)	H22A—C22—H22C	109.5
C9—C8—C7	119.0 (2)	H22B—C22—H22C	109.5
C9—C8—H8	120.5	C23—N3—C21	119.7 (3)
C7—C8—H8	120.5	C23—N3—C22	121.6 (4)
C8—C9—C10	120.3 (3)	C21—N3—C22	118.5 (4)
C8—C9—H9	119.9	O5—C23—N3	123.8 (3)
C10—C9—H9	119.9	O5—C23—H23	118.1
N2—C10—C9	122.1 (2)	N3—C23—H23	118.1
N2—C10—H10	119.0		
			
O3—Cu1—N1—C1	−11.6 (2)	C8—C7—C11—N2	−0.1 (4)
N2—Cu1—N1—C1	178.2 (2)	C6—C7—C11—N2	179.4 (2)
O1W—Cu1—N1—C1	85.2 (2)	C8—C7—C11—C12	−179.5 (2)
O3—Cu1—N1—C12	170.25 (17)	C6—C7—C11—C12	0.0 (3)
N2—Cu1—N1—C12	0.02 (16)	C1—N1—C12—C4	1.3 (4)
O1W—Cu1—N1—C12	−92.91 (17)	Cu1—N1—C12—C4	179.64 (18)
O2—Cu1—N2—C10	6.0 (2)	C1—N1—C12—C11	−178.4 (2)
O3—Cu1—N2—C10	132.5 (3)	Cu1—N1—C12—C11	0.0 (3)
N1—Cu1—N2—C10	−179.5 (2)	C3—C4—C12—N1	−0.6 (4)
O1W—Cu1—N2—C10	−89.2 (2)	C5—C4—C12—N1	−179.8 (2)
O2—Cu1—N2—C11	−174.44 (16)	C3—C4—C12—C11	179.0 (2)
O3—Cu1—N2—C11	−48.0 (4)	C5—C4—C12—C11	−0.1 (4)
N1—Cu1—N2—C11	−0.01 (15)	N2—C11—C12—N1	0.0 (3)
O1W—Cu1—N2—C11	90.35 (16)	C7—C11—C12—N1	179.4 (2)
O3—Cu1—O2—C19	21.4 (2)	N2—C11—C12—C4	−179.7 (2)
N2—Cu1—O2—C19	−169.2 (2)	C7—C11—C12—C4	−0.3 (3)
O1W—Cu1—O2—C19	−75.9 (2)	O1—C13—C14—C15	4.5 (5)
O2—Cu1—O3—C20	−26.0 (2)	O1—C13—C14—C19	−175.7 (3)
N1—Cu1—O3—C20	160.4 (2)	C19—C14—C15—C16	0.5 (4)
N2—Cu1—O3—C20	−152.3 (3)	C13—C14—C15—C16	−179.7 (3)
O1W—Cu1—O3—C20	69.7 (2)	C14—C15—C16—C17	−0.9 (5)
C12—N1—C1—C2	−1.1 (4)	C15—C16—C17—C18	0.8 (5)
Cu1—N1—C1—C2	−179.2 (2)	C16—C17—C18—C19	−0.3 (4)
N1—C1—C2—C3	0.3 (4)	C16—C17—C18—C20	−178.7 (3)
C1—C2—C3—C4	0.3 (4)	Cu1—O2—C19—C14	168.07 (16)
C2—C3—C4—C12	−0.2 (4)	Cu1—O2—C19—C18	−12.3 (3)
C2—C3—C4—C5	178.9 (3)	C15—C14—C19—O2	179.7 (2)
C12—C4—C5—C6	0.8 (4)	C13—C14—C19—O2	−0.1 (4)
C3—C4—C5—C6	−178.3 (3)	C15—C14—C19—C18	0.1 (4)
C4—C5—C6—C7	−1.1 (4)	C13—C14—C19—C18	−179.7 (2)
C5—C6—C7—C11	0.7 (4)	C17—C18—C19—O2	−179.8 (2)
C5—C6—C7—C8	−179.9 (3)	C20—C18—C19—O2	−1.5 (4)
C11—C7—C8—C9	0.0 (4)	C17—C18—C19—C14	−0.2 (3)
C6—C7—C8—C9	−179.4 (3)	C20—C18—C19—C14	178.1 (2)
C7—C8—C9—C10	0.0 (4)	Cu1—O3—C20—O4	−162.1 (2)
C11—N2—C10—C9	−0.2 (4)	Cu1—O3—C20—C18	19.6 (3)
Cu1—N2—C10—C9	179.35 (19)	C17—C18—C20—O4	−2.0 (4)
C8—C9—C10—N2	0.1 (4)	C19—C18—C20—O4	179.7 (2)
C10—N2—C11—C7	0.2 (3)	C17—C18—C20—O3	176.3 (2)
Cu1—N2—C11—C7	−179.38 (18)	C19—C18—C20—O3	−2.0 (4)
C10—N2—C11—C12	179.6 (2)	C21—N3—C23—O5	−0.7 (6)
Cu1—N2—C11—C12	0.0 (3)	C22—N3—C23—O5	−176.8 (4)

### Hydrogen-bond geometry (Å, °)

**Table:**

*D*—H···*A*	*D*—H	H···*A*	*D*···*A*	*D*—H···*A*
O1W—H1WB···O4^i^	0.85	1.91	2.741 (3)	167
O1W—H1WA···O5	0.85	1.96	2.794 (3)	167

Symmetry codes: (i) −*x*+1, −*y*, −*z*+1.
